# Air pollution and detrimental effects on children’s brain. The need for a multidisciplinary approach to the issue complexity and challenges

**DOI:** 10.3389/fnhum.2014.00613

**Published:** 2014-08-12

**Authors:** Lilian Calderón-Garcidueñas, Ricardo Torres-Jardón, Randy J. Kulesza, Su-Bin Park, Amedeo D’Angiulli

**Affiliations:** ^1^Department of Biomedical Sciences, The Center for Structural and Functional Neurosciences, University of MontanaMissoula, MT, USA; ^2^Centro de Ciencias de la Atmósfera, Universidad Nacional Autonoma de MexicoMexico City, Mexico; ^3^Auditory Research Center, Lake Erie College of Osteopathic MedicineErie, PA, USA; ^4^Neuroscience, NICER Lab (Neuroscience of Imagery Cognition and Emotion Research Lab), Carleton UniversityOttawa, ON, Canada

**Keywords:** urban children, air pollution, cognition, brain volumetric changes, white matter hyperintensities, cytokines, Alzheimer, Parkinson

## Abstract

Millions of children in polluted cities are showing brain detrimental effects. Urban children exhibit brain structural and volumetric abnormalities, systemic inflammation, olfactory, auditory, vestibular and cognitive deficits v low-pollution controls. Neuroinflammation and blood-brain-barrier (BBB) breakdown target the olfactory bulb, prefrontal cortex and brainstem, but are diffusely present throughout the brain. Urban adolescent Apolipoprotein E4 carriers significantly accelerate Alzheimer pathology. Neurocognitive effects of air pollution are substantial, apparent across all populations, and potentially clinically relevant as early evidence of evolving neurodegenerative changes. The diffuse nature of the neuroinflammation and neurodegeneration forces to employ a weight of evidence approach incorporating current clinical, cognitive, neurophysiological, radiological and epidemiological research. Pediatric air pollution research requires extensive multidisciplinary collaborations to accomplish a critical goal: to protect exposed children through multidimensional interventions having both broad impact and reach. Protecting children and teens from neural effects of air pollution should be of pressing importance for public health.

## Introduction

In epidemiological studies, clean air has been linked to children’s health and wellbeing (Newman et al., 2013; Amato et al., 2014; Barbieri et al., 2014; Liu and Lewis, 2014; Perera et al., 2014; Tang et al., 2014). Although most of the human studies have shown an associative link, experimental animal studies measuring exposure to specific components have shown a causal relationship between air pollution and an array of detrimental effects. Around the world, several metropolitan areas—but especially megacities (defined as areas of continuous urban development of over 10 million people, Kotkin and Cox, 2014)—now exceed the standards for air pollutants. Consequently, millions of children are at risk for or are already showing adverse short and long-term health outcomes, which include some of the most detrimental effects on brain development (Calderón-Garcidueñas et al., 2008; Brook et al., 2010; Guxens and Sunyer, 2012; Becerra et al., 2013). However, for the most part, current research and policy efforts link air pollution to respiratory and cardiovascular disease (Brook et al., 2010), and the effects on children’s central nervous system (CNS) are still not broadly recognized. As a result, wide reaching public health initiatives targeting pediatric populations are still considered premature or unwarranted. One of the goals of this review is to show that contrary to a hesitant approach, there is enough evidence supporting the perspective that the effects of air pollution on brains of children and teens ought to be key public health targets.

In this paper, we briefly review current air pollutant standards, followed by epidemiological, clinical, and pathology studies associating air pollution exposures on children’s brains concerning cognitive abilities, neurodevelopmental and neurodegenerative diseases. This overview puts forward common denominators for the mechanistic pathways linking air pollution to negative effects on the developing brain. Then, we turn to the outstanding challenges including the issues of how to formulate strategies to study *clinically healthy children exposed to air pollutants*, how to establish the links with the current mainstream concepts of cognition and neurodevelopment on one hand, and systemic inflammation, neuroinflammation, structural and volumetric brain changes and neurodegeneration on the other, followed by the ultimate goal to protect exposed children.

The present perspective indicates the need of a multidisciplinary approach, not only to address the issue complexity and challenges, but also to make developmental, behavioral and clinical researchers and practitioners aware of the wide spectrum of air pollution effects and the potential impact on their daily practice.

## Air pollutants: what are they? Where are they?

In the US, the 1970 amendments to the Clean Air Act required the Environmental Protection Agency (EPA) to set National Ambient Air Quality Standards (NAAQS) for certain pollutants known to be hazardous to human health. EPA identified six criteria pollutants: ozone, carbon monoxide, particulate matter (PM), sulfur dioxide, lead, and nitrogen oxide and set the standards as a function of the characteristics and their potential health and welfare effects. In the US alone, more than 103 million people are exposed to PM concentrations above the standards, while 123 million are exposed to ozone. The two fractions of PM predominantly implicated in CNS effects are PM_2.5_ (particles with a diameter <2.5 μm) and ultrafine PM (UFPM) (particles with a diameter <100 nm). Most Outdoor PM_2.5_, were generated from tailpipe and brake emissions from mobile sources, residential fuel combustion, power plants, wildfires, oil refineries, and metal processing facilities. The primary contributors to UFPM are tailpipe emissions from mobile sources.

Indoor air pollutants, including tobacco smoke, emissions from cook stoves, mycotoxins, plasticizers, flame retardants, and pesticides also represent a major source of harmful substances. Indoor air quality in schools is a major issue as the presence of mold, poor air quality, close proximity to major highways, and contaminated playgrounds can result in serious health problems (Everett-Jones et al., 2010; Sampson, 2012). Moreover, there are major disparities in indoor air pollution exposures related to socio-economic status (SES): the lower the SES, the higher indoor exposures (Adamkiewicz et al., 2011). Children are also exposed to manufactured nanoparticles (NPs) (>100 nm) in many consumer products including food, sunscreens and toothpaste (Linsinger et al., 2013).

## Animal models of outdoor air pollution components and brain effects

The complexity of the urban atmosphere makes it very difficult to establish a direct association of CNS effects with specific air pollutants in humans. Fortunately, animal models exposed to air pollutant components such as ozone, PM, diesel NPs, endotoxins, etc., have contributed a good deal to our understanding of the potential mechanisms acting upon the CNS. Depending on the pollutant component, doses, exposure protocol, age and gender, health status, etc., the detrimental effects range from endothelial dysfunction, breakdown of the blood-brain-barrier (BBB; Levesque et al., 2011), neuroinflammation (Fonken et al., 2011), formation of free radicals and oxidative stress (Guo et al., 2012), dopaminergic neuronal damage, RNA and DNA damage, to the identification of early hallmarks of Alzheimer and Parkinson’s diseases (Brun et al., 2012).

In spite of the complexity, the evidence conclusively showed in animals that prenatal exposure to either one or a combination of criteria pollutants caused permanent changes in neurotransmitters and altered brain development, most commonly resulting in long-term deficits in functions associated with one or more memory systems (Takahashi et al., 2010; Fonken et al., 2011; Umezawa et al., 2012; Schröder et al., 2013).

## Children’s systemic and brain effects of air pollution

**Figure 1:**
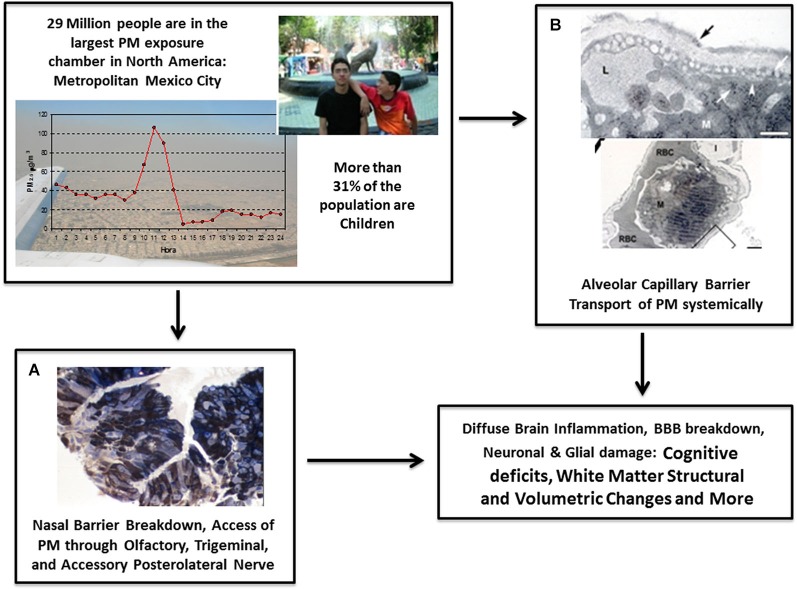
**Main mechanisms involved in brain damage associated with PM include the breakdown of nasal and olfactory pathways **(A)** and the alveolo-capillary pathway **(B)****. All critical stages and effects of the biological pathways linked with **(A)** and **(B)** are shown in Figure 2. Through these pathways, PM produces systemic inflammation and travels around the body to ultimately cause pathological alterations including neuroinflammation, breakdown of the BBB, neuronal and glial damage, changes in white matter structure and volume, decline in cognitive abilities, auditory and vestibular impairment, and further deficits. **(A)** Nasal biopsy of 10 year-old Mexico City (MC) boy showing fine PM in the cytoplasm of epithelial cells stained with 1 μm toluidine blue. **(B)** Passage of PM through the alveolar capillary barrier in a Metropolitan MC young resident.

**Figure 2:**
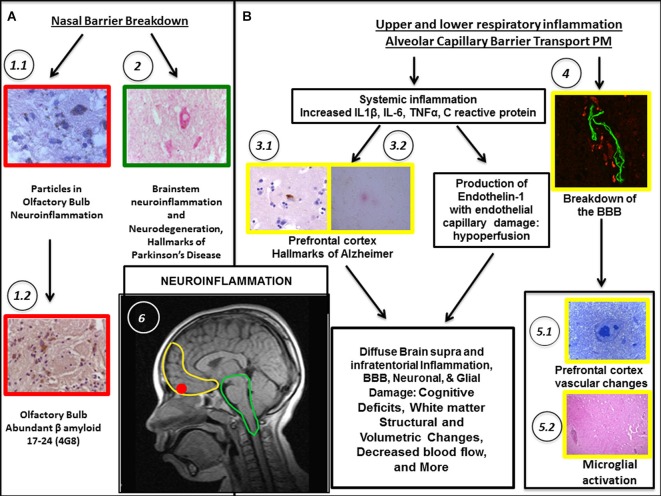
**Biological pathways associating PM exposure with the breakdown of nasal and olfactory pathways **(A)** and upper/lower respiratory tract inflammation along the passage of PM through the alveolo-capillary barrier **(B)** are shown**. Localization in the brain (see **B-6**) is coded by the color of the outline frames of the panels: Yellow = prefrontal cortex; Red = Olfactory bulb; Green = Brainstem; Black and Arrows = stages along the pathways. Neuroinflammation is the common denominator. In **(A)**, nasal breakdown allows PM to directly access the brain. **(A)** pathway shows accumulated PM particles in the olfactory bulb. **(A-1.1)** Fourteen year-old MC boy with abundant particulate material in neurons in the glomerular region. **(A-1.2)** Accumulation of beta β amyloid, a hallmark of Alzheimer’s disease (AD). **(A-2)** Inflammation and degenerative changes are significant in the brainstem. For example, accumulation of α synuclein in dopaminergic pigmented neurons in an 11 year-old MC girl is a hallmark of Parkinson’s disease. In **(B)**, the extensive respiratory tract inflammation and the passage of ultrafine PM through the alveolar capillary barrier allows PM to access the body resulting in systemic inflammation. The increased production of endothelin 1, a potent vasoconstrictor results in vasoconstriction and cerebral hypoperfusion. The BBB (see especially **B-4**) is damaged and triggering of autoimmune responses directly damages neural components. **(B-3.1)** Frontal cortex in a 15 year-old boy. Abnormal tau protein-a marker of AD- both in the neuronal body and in neurites. **(B-3.2)** Frontal cortex in a 17 year-old boy. A diffuse amyloid plaque –a marker of AD–(red product) is seen surrounded by glial cells. **(B-4)** Frontal white matter blood vessel in a 20 year-old MC male shows BBB breakdown. **(B-5.1)** Young 15 month-old MC dog, frontal white matter abnormal arteriole. **(B-5.2)** Entorhinal area perivascular inflammation in a 22 year-old female from MC. **(B-6)** Sagittal view of MRI of a male MC resident aged 10.

The detrimental developmental effects in animals are conceptually related to or even mirror the effects that might be expected and are actually observed in children. Consequently, it is reasonable and plausible to assume a fundamental continuity underlying the processes that impact the developing brain, in human or animal. In this section, selected possible mechanisms of the action of PM air pollution shown in Figures 1, 2 are specifically linked to key evidence found in Mexico City (MC) children.

Urban residents exhibit extensive respiratory inflammation that targets the nasal epithelium (Calderón-Garcidueñas et al., 1992). Severe mucosal changes translate in a breakdown of the nasal epithelial barrier facilitating the passage of xenobiotics to the systemic circulation and the brain, including PM both fine and ultrafine. The pulmonary damage is equally severe (Calderón-Garcidueñas et al., 2003), and boys are more affected than girls, likely because of longer daily outdoor activities (Villarreal-Calderón et al., 2002). Systemic inflammation and endothelial dysfunction with high production of endothelin-1 (ET-1) are also of importance. High concentrations of powerful inflammatory mediators such as interleukin-1β, tumor necrosis factor alpha (TNFα), and interleukin 6 (IL6) for which brain endothelial cells have receptors are present in the exposed pediatric populations. High ET-1 plasma concentrations, a very potent vasoconstrictor, negatively impact the brain microvasculature, resulting in hypoperfusion and ET-1 concentrations are directly associated with PM_2.5_ exposures (Calderón-Garcidueñas et al., 2007).

Post-mortem neuropathology studies in children with accidental deaths comparing high vs. low pollution exposures revealed that 40% of urban children exhibited frontal tau hyperphosphorylation with pre-tangle material and 51% had Aβ_42_ diffuse plaques compared with 0% in controls (Calderón-Garcidueñas et al., 2012a). Hyperphosphorylated tau (HPτ) and Aβ_42_ plaques are hallmarks of Alzheimer’s disease (AD; Braak and Del Tredeci, 2011). Of utmost importance for this review, children with the Apolipoprotein E allele 4 (a well-known risk factor for AD) had greater HPτ and diffuse Aβ plaques vs. E3 carriers, suggesting that genetic factors could make a significant portion of the exposed population more prone to accelerating their AD pathology (Calderón-Garcidueñas et al., 2012a). Arteriolar and capillary vascular changes with a diffuse breakdown of the BBB are at the core of the pathology in exposed children’s brains. The changes are significant in the olfactory bulb and the prefrontal white matter, but can also be found in every lobe and in the brainstem (Calderón-Garcidueñas et al., 2013).

*Clinically healthy children* from MC selected by stringent criteria including the absence of known risk factors for cognitive or neurological deficits, exhibited structural, neurophysiological and cognitive detrimental effects compared to matched SES, gender, age and mother’s IQ low pollution exposed children (Calderón-Garcidueñas et al., 2008, 2011a). The cognitive deficits in MC children matched the MRI volumetric changes in their right parietal and bilateral temporal areas (Calderón-Garcidueñas et al., 2012b). Complex modulation of cytokines and chemokines influences children’s CNS structural and volumetric responses and cognitive correlates resulting from environmental pollution exposures. MC children performed more poorly across a variety of cognitive tests in comparison to the control (Calderón-Garcidueñas et al., 2008, 2011a,b).

A number of abnormalities within the auditory brainstem nuclei have been identified in children exposed to severe air pollution. Specifically, the neuronal cell bodies within the medial superior olive (MSO) are significantly smaller and more round than those in age-matched control brains (Calderón-Garcidueñas et al., 2011b). This finding is important because the MSO has clear roles in localization of sound sources, encoding temporal features of sound and likely plays an important role in brainstem encoding of speech. Integrity of the auditory brainstem nuclei can be accessed through a number of noninvasive techniques, such as brainstem auditory evoked potentials (BAEPs), otoacoustic emissions, speech recognition tasks and listening in background noise. Incidentally, similar morphological alterations were observed in autistic children (Kulesza and Mangunay, 2008; Kulesza et al., 2011) and correlated with abnormal brainstem reflexes (Lukose et al., 2013). Urban children have delayed central conduction time of brainstem neural transmission, resulting in increased risks for auditory and vestibular impairments and altered speech recognition abilities (Calderón-Garcidueñas et al., 2011b).

Based on the described evidence, it is reasonable to argue that air pollution exposed children experience a chronic, intense state of oxidative stress and exhibit an early brain imbalance in genes involved in inflammation, innate and adaptive immune responses, cell proliferation, necrosis and apoptosis (Calderón-Garcidueñas et al., 2012a, 2013). Neuroinflammation, endothelial activation, the significant heterogeneity of endothelial cells in CNS microvessels, and the BBB breakdown contribute to cognitive impairments, pathogenesis and pathophysiology of neurodegenerative states (Jian et al., 2012; Roher et al., 2012; Paul et al., 2013).

## Children’s outcomes associated with the impact of air pollution

The associations between cognition and urban pollution have been established in cities like Boston, where black carbon, a marker for traffic PM, predicted decreased cognitive function across assessments of verbal and nonverbal intelligence and memory in 9 year-olds (Suglia et al., 2008). Although genetic factors play a key role in CNS responses (as evidenced by the acceleration of neurodegenerative pathology in children carrying an APOE 4 allele), studies such as the abovementioned ones in Boston and others, sketch a complex scenario where air pollution and SES can influence neural development and cognition along with known factors such as psychosocial stress and poor nutrition, thereby influencing and determining mental health, academic achievements and overall life performance (Siddique et al., 2011; Becerra et al., 2013). It is critical to point out that although SES is an additive independent risk factor, in several of the studies conducted not only in megacities such as New York City, Beijing, Sao Paulo, Los Angeles, but also in smaller metropolitan areas including Boston, Cincinnati, and Barcelona that we have reviewed earlier (Newman et al., 2013; Amato et al., 2014; Barbieri et al., 2014; Liu and Lewis, 2014; Perera et al., 2014; Tang et al., 2014), the effects of *outdoor* air pollution on children’s brain did not vary interactively with low SES. Thus, outdoor air pollution effects are not a concern for just underprivileged populations although the fact that belonging to the lower end of the socioeconomic spectrum is very likely to aggravate detrimental health effects.

Although the direct and indirect influences of air pollution on several developmental outcomes are not fully understood, psychiatrists, clinical psychologists and allied mental health and pediatric professionals have a critical role to play in identifying the potential associations between exposure and behavioral issues. There are several emerging trends of evidence suggesting that air pollution may be associated with an array of atypical neurocognitive and behavioral changes in children and teens which are of legitimate public health concern and call for prediction and prevention of early environmental health risk factors (Borges et al., 2011; Haynes et al., 2011; Liu, 2011; Liu and Lewis, 2014).

An intriguing association has been identified recently between autism spectrum disorder including attention deficit hyperactive disorders (ADHD) and particle air pollution (Larsson et al., 2009; Zhang et al., 2010; Siddique et al., 2011; Becerra et al., 2013; Volk et al., 2013). Risk factors related to PM include maternal second and third hand smoke exposure, residency during gestation at the highest quartile of exposure to traffic-related air pollution, condensation on windows (a proxy for low rate of ventilation in homes) and polyvinyl chloride (i.e., indoor airborne phthalates) flooring, especially in the bedroom of parents. Interestingly, airway symptoms of wheezing and physician-diagnosed asthma were also associated with autism spectrum disorder 5 years later (Larsson et al., 2009). Since these associations are linking autism and ADHD with environmental variables, they warrant wider knowledge translation by and among the developmental, behavioral and clinical researchers and practitioners.

There are now also a handful of studies examining the effects of psychosocial stress and air pollution together on asthma; some suggest they act synergistically, whereas others find a more complex interaction where the socioeconomic and environmental conditions co-modulate the respiratory outcomes (for a review see Wright, 2011). These studies and the evolving asthma literature that speaks to the interactions between disease activity, psychosocial stress, learning disabilities, cognition and air pollution (Caldera-Alvarado et al., 2013) are further evidence of the more complex interactions impacting the CNS and the need for a multidisciplinary approach in the management of developmental, behavioral and cognitive problems in children at risk.

The reviewed evidence of brain, neurocognitive and behavioral detrimental outcomes associated with air pollution, collectively suggests substantive effects that may have long-term clinical repercussions in terms of degenerative diseases (Calderón-Garcidueñas et al., 2013). Given the social and economic burden of accelerated aging in our society, whose far-reaching ramifications are simply incalculable (see National Institute of Aging, National Institutes of Health and World Health Organization, 2001), a multidisciplinary approach aiming at screening target school populations that are most at risk, would seem a rather cost-effective and most beneficial public health strategy. Strong support for the need of neurocognitive and behavioral screening in the targeted risk populations of children comes from a growing psychological and epidemiological literature suggesting evidence of suboptimal cognitive functioning across the developmental span in *clinically healthy children* (Calderón-Garcidueñas et al., 2012b; Guxens and Sunyer, 2012). Importantly, a significant proportion of urban schools are situated near major traffic-related air pollution sources (Amram et al., 2011), and cognitive outcomes may be partly associated with air pollution levels around schools (Mohai et al., 2011).

## Limitations of current research and future directions: need for a multidisciplinary approach and children’s public health priority agenda

Air pollution effects on the developing brain may vary along a continuum from minor, subtle subclinical deficits in cognitive functioning to significant cognitive deficits that are identified readily by parents and/or teachers. The detrimental effects may also worsen with the age of the child, thus selected neurocognitive tools ought to be useful for measuring longitudinal studies across educational backgrounds and predicting overlaps in the functional areas and tests affected. Complex cognitive responses that may be affected include: attention and short-term memory, information processing speed and executive function, verbal abstraction, and visuospatial and motor skills. Deficits in auditory and vestibular responses and sound localization could also be expected, along with olfaction deficits. Most of the neuroimaging studies already mentioned (specifically using techniques such as Electroencephalography/Event-Related Potentials, BAEPs, structural and functional Magnetic Resonance Imaging, and Magnetic Resonance Spectroscopy) conducted in clinical and preclinical settings have all been reported to show a gradient of effects. However, one area of limitation is our fragmentary knowledge behind the pathology and the mechanisms of neurodevelopmental and neurodegenerative disorders that are exhibiting overlapping expressions for several of the effects identified in this review. Future studies need to be designed so that this limitation can be overcome.

Consistent with these observations, the National Institute of Environmental Health Sciences/National Institute of Health panel on outdoor air pollution indicated cognitive and neuropsychological (and possibly neuroimaging) screenings of children as one of the priority for future research advocating a multidisciplinary collaborative approach wherein brain-related development testing would have a prominent role (Block et al., 2012).

It seems relatively straightforward that health professionals, behavioral scientists, psychologists and psychiatrists should each have a responsibility to address the particular issues associated with air pollution in the measure and modality in which the individuals are impacted. Furthermore, the diffuse nature of the neuroinflammation and the evolving neurodegenerative changes observed in exposed children obligates us not to rely on a single study or measure but rather to employ a weight of evidence approach incorporating current clinical, neurophysiological, radiological and epidemiological research as well as the results of animal exposure studies to a single pollutant/mixtures/or pollutant components. Inflammatory biomarkers play a key role in the identification of children with positive volumetric and cognitive responses to their lifelong pollutant exposures (Calderón-Garcidueñas et al., 2012b). Since neuroinflammation/vascular damage/neurodegeneration go hand in hand (Calderón-Garcidueñas et al., 2013), establishing the definition of inflammatory/endothelial dysfunction biomarkers regarding the association between brain growth and developmental behavioral as well as psychological outcomes are urgently needed.

If the evidence is so convincing, why do not we lower pollution standards instead of launching expensive public health initiatives? Based on the current evidence and history, it seems extremely improbable that a global issue such as air pollution reduction will find a prompt consensus on decisive policy action towards better standards and their execution. The evidence accumulated so far clearly indicates that the neurocognitive effects of air pollution are substantive, they are apparent across all populations (not just the disadvantaged ones), and most importantly, the observed neurocognitive impairments are potentially and clinically relevant as an early indicator of evolving neurodegenerative precursors. Our ultimate goal should be to protect severely exposed children in large urban areas through multidimensional interventions yielding both impact and reach (i.e., on cognitive/behavioral, family participation, and modifiable lifestyle factors such as diet and micronutrient supply). One beneficial and cost-effective strategy for achieving those objectives is to have air pollution brain effects as key public health targets, and monitor the pediatric populations that are most at risk through preventative screening programs.

## Conflict of interest statement

The reviewer Dr. Lipina declares that, despite having collaborated with the authors, the review process was handled objectively. The authors declare that the research was conducted in the absence of any commercial or financial relationships that could be construed as a potential conflict of interest.
